# Rat Calvarial Defect Models in Bone Regeneration Research: A Narrative Review of Critical Size and Micro-Computed Tomography Outcome Reporting

**DOI:** 10.3390/ani16193142

**Published:** 2026-10-07

**Authors:** Klemen Aleš Pilih, Uroš Kovačič

**Affiliations:** 1 Department of Traumatology, General Hospital Slovenj Gradec, Gosposvetska Cesta 1, 2380 Slovenj Gradec, Slovenia; 2 Institute of Pathophysiology, Faculty of Medicine, University of Ljubljana, Zaloška Cesta 4, 1000 Ljubljana, Slovenia; uros.kovacic@mf.uni-lj.si

**Keywords:** calvarial defect, critical-size defect, bone regeneration, micro-computed tomography, rat model, 3Rs, reporting standards

## Abstract

The rat calvarial defect model supports the preclinical evaluation of bone-regenerative approaches relevant to craniofacial reconstruction, including recombinant human bone morphogenetic protein-2 (rhBMP-2) and bone substitutes. However, its invasive nature requires study-specific harm–benefit assessment and application of the 3Rs. This narrative review used a structured PubMed search to examine spontaneous healing in defects smaller than 5 mm and μCT outcome reporting in untreated or control defects. Twenty-four studies of sub-5 mm defects and 45 μCT studies were included. Complete spontaneous healing was not reported in the sub-5 mm studies; only two μCT studies reported regeneration of at least 50% according to their respective outcome measures. Healing generally increased with longer observation periods and smaller defects, although methodological heterogeneity limited comparisons. Inconsistent reporting of acquisition parameters, segmentation thresholds, regions of interest, and numerical outcomes restricted reproducibility and data reuse. Within this PubMed-indexed evidence base, the findings support 5 mm defects as a practical model of incomplete spontaneous healing and indicate that smaller defects also do not reliably heal completely within the observation periods studied. These findings do not directly establish clinical benefit. Future studies should justify the least burdensome model capable of answering the research question, implement replacement, reduction, and refinement, and report standardized bone volume fractions, relevant measures of defect bridging, reproducible imaging methods, and reusable numerical data.

## Simple Summary

Rat skull defects are widely used to test methods intended to improve bone healing, but researchers need reliable information about which defects heal naturally and how healing should be measured. We reviewed published studies of untreated rat skull defects, focusing on defect size and three-dimensional X-ray imaging used to measure new bone. Defects smaller than 5 mm often healed only partly, suggesting that even these smaller defects may be critical-sized under certain experimental conditions. Reported healing varied greatly between studies because animal characteristics, surgeries, observation times, imaging settings, and outcome definitions were not consistent. Many studies also failed to provide numerical data that could be reused. Future studies should use the least severe defect that can answer the research question, justify animal numbers and procedures, and report imaging methods and numerical outcomes clearly. Better planning and reporting can improve reproducibility, reduce unnecessary repetition, and increase the knowledge gained from each animal.

## 1. Introduction

Bone has a remarkable capability to regenerate without scar tissue formation [1]. However, when regenerative capabilities are exhausted, non-union or delayed union may form. Therapy of such bony malunions can nowadays still present as clinically challenging [2], often requiring surgical intervention. However, bio-engineering development has brought about several new treatment options [3]. With such a plethora of therapeutic interventions, animal models that enable evaluation of different treatment strategies had to be developed. Gomes and Fernandes [4] have reviewed the use of rodent models in bone-related research. They state that these models are advantageous due to the ease of access, easy handling, low cost, and vast amount of background data related to their widespread use. Therefore, it comes as no surprise that O’Loughlin et al. [5], when they reviewed the use of animal models in fracture repair studies in 2008, found that rodent models represented more than half of the animal models used in the last ten years.

While long bones grow and heal mostly by endochondral ossification [6], flat bones depend on intramembranous ossification [7]. As a result, two groups of fracture/defect animal models are used: long-bone models (mostly femur and tibia) and flat-bone models (mostly the calvaria of the skull). The practical applicability of long-bone models lies in treating major bone defects following trauma or tumor resection [8,9]. In contrast, flat-bone models mainly serve the field of cranio-maxillofacial surgery [10].

The term “critical-size defect”, indicating a bone defect that does not heal spontaneously, was coined by Schmitz and Hollinger in 1986 [11] and was later redefined by Gosain et al. [12] to “the size of a defect that will not heal over the duration of the study”. A bone defect of a smaller size that heals spontaneously during the study should, by analogy, be termed a “subcritical-size defect”. Since their introduction, “critical-size defect” models have gained popularity in the testing of different methods of enhancing bone repair [13,14,15,16,17,18,19,20,21]. However, in the last decade, there has been a lack of literature reviews on the topic of (sub-)critical bone defect healing.

In addition, an appropriate methodology to measure bone healing in experimental animal models had to be updated in accordance with modern technological solutions. Computerized histomorphometry analysis has been the first upgrade from semiquantitative or even descriptive histology. Recently, micro-computed tomography (μCT) has emerged as a gold-standard tool, enabling the display and analysis of bone morphology down to the level of an individual bone trabecula. μCT is advantageous because it can be conducted in vivo and thereby allows for longitudinal observations of the bone-healing progress over the life of the same animal. Due to its apparent advantages, μCT has gained widespread use either as a solo diagnostic method or complemented by histomorphometry in rodent models of bone healing [22].

The purpose of our study was to review the current literature on the rat calvarial critical defect model from two aspects:

1. Based on a review of the literature, performed a decade ago [23], it has been established that 5 mm is the critical size for the rat calvaria defect model. However, this could not be claimed with certainty for defects of lesser sizes due to an inadequate volume of relevant data at the time of the previous review. We aimed to review the literature to determine whether additional conclusions about the (sub-)critical size of calvarial defects can be made after a decade of scientific contribution to the subject.
2. Since μCT has become an established method of bone-healing analysis, our goal was to review how widely present it is. In addition, we aimed to identify the optimal ways of reporting results of defect healing based on μCT image analysis. If possible, our goal was to make recommendations on μCT use and the methods of reporting the results.

## 2. Materials and Methods

### 2.1. Narrative Review Approach

This narrative review incorporated a structured search component. The literature base was assembled from PubMed and divided into two subsets: studies addressing the spontaneous healing of defects smaller than 5 mm and studies using μCT to quantify new bone formation in untreated or control defects. In intervention studies, only untreated or control defects were included. PubMed was selected for its biomedical scope and controlled MeSH vocabulary, which supported a transparent, manageable search within the time and resources available for this narrative review. The included literature represents a PubMed-indexed subset, not an exhaustive systematic collection or a statistically representative sample of all relevant research.

This review was not prospectively registered, and no formal risk-of-bias tool was applied. Because the studies differed in animal characteristics, defect designs, observation periods, surgeries, μCT acquisition and analyses, and outcome definitions, the evidence was synthesized narratively. Grouped quantitative data are presented only as exploratory descriptive context, not as pooled effect estimates.

### 2.2. Review Strategy

PubMed was searched using MeSH terms and supplementary free-text terms. The study-pool query was “Rats” [MeSH Terms] AND “Skull” [MeSH Terms] NOT “Facial Bones” [MeSH Terms] AND (“Bone Regeneration” [MeSH Terms] OR “Osteogenesis” [MeSH Terms]), which yielded 1229 records. Subsequent queries were combined with this study-pool query using AND. One reviewer (K.A.P.) screened records and extracted data; uncertain decisions were resolved through discussion with the second author (U.K.).

For defects smaller than 5 mm, free-text terms describing noncritical or subcritical defects and diameter terms from 2.0 to 4.9 mm were used. After duplicate removal and screening, 24 studies were included in the subcritical-size synthesis (Table 1).

For the μCT synthesis, “X-Ray Microtomography” [MeSH terms] was combined with the study-pool query and yielded 189 records. After title and abstract screening, 142 reports underwent full-text assessment; 97 were excluded (48 lacked reusable numerical μCT outcomes, 34 had material in every nominal control defect, and 15 used an alternative model), leaving 45 studies (Table 2). Corresponding authors were contacted when numerical values were unavailable. If no usable response was obtained, values were digitized from calibrated published figures and checked against graph axes and sample descriptions. Where the sample size or variability measure remained uncertain, the more conservative assumption was used and flagged in the Supplementary Tables. Uncertain extracted counts and variability types are flagged in Tables S1 and S2. Variability is retained in its extracted form; an unknown measure is not relabeled as SEM, and an assumed count is not used to estimate precision.

**Figure 1:**
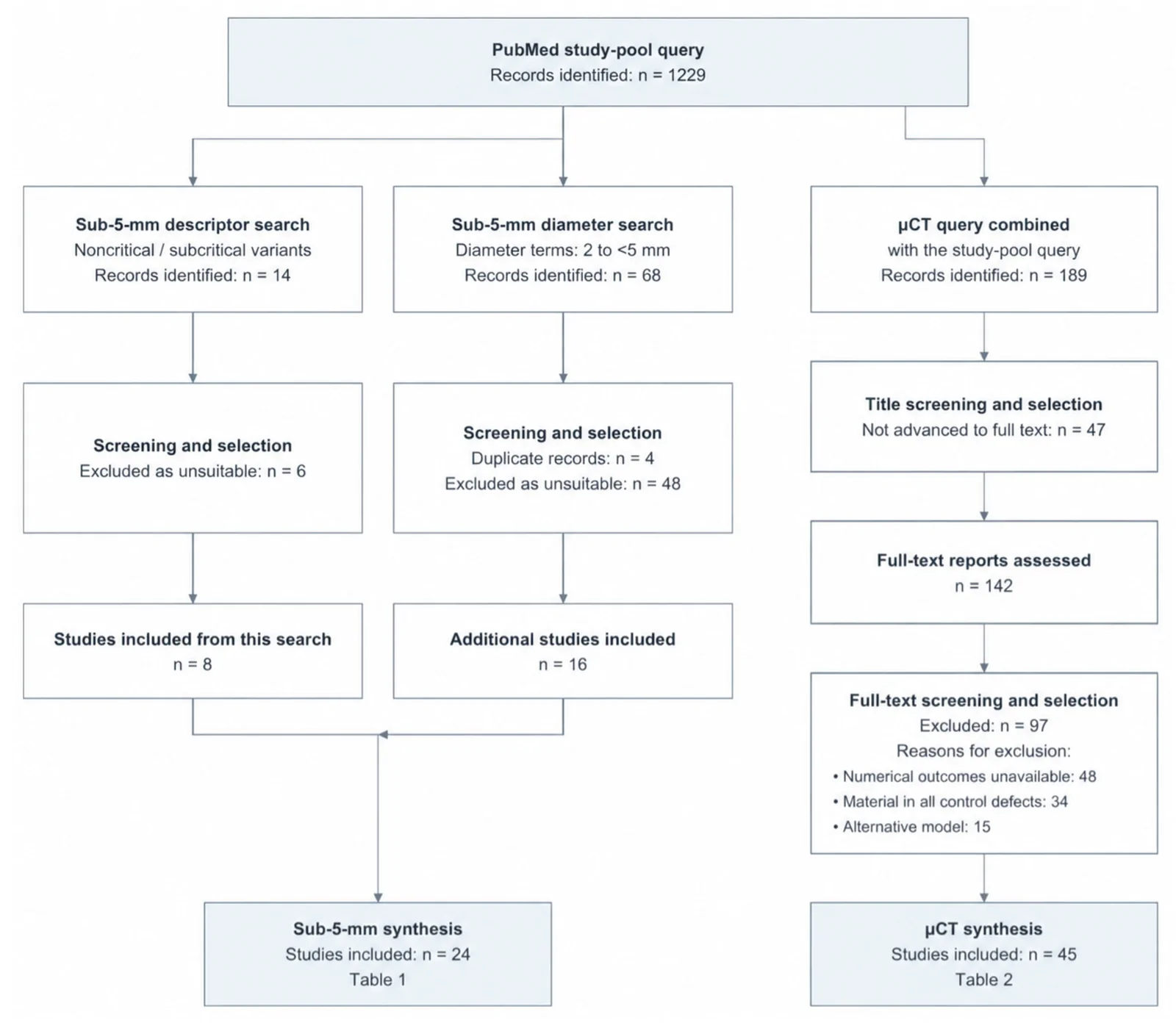
Study selection diagram for two narrative review subsets.

Figure 1 summarizes the documented selection counts.

### 2.3. Exploratory Grouped Quantitative Summaries

Exploratory grouped summaries were retained because comparison across several studies can reveal patterns not evident in individual reports [24]. Studies reporting comparable percentage-based BV/TV outcomes were grouped by defect diameter (<5, 5, 6, 7, 8, and 9 mm) and observation period (1, 2, 3, 4, 6, 8, 10, 12, and 20–30 weeks). The study-level means and variability are displayed without treating the groups as pooled effect estimates.

Groups containing three or fewer studies were retained only to show the available range and were not interpreted as stable benchmarks. Values digitized from figures or based on uncertain sample sizes or variability measures were flagged and interpreted cautiously. Where standard deviation and sample size were available, the standard error of the mean was calculated as SD/√n solely to display variability on a common descriptive scale [25].

**Table 1:** Studies included in subcritical-size defect review and their essential characteristics.

Study [Ref.]	Authors	Strain	Age	Rats/ Time	Defects/Rat	Obs.	Diameter	Time	Closure	New Bone Formation (%) and Method	Other Measurements
1 [26]	Diallo et al.	W	12 w	6	3	12	4 mm	60 d	N/A	N/A	Radiopaque volume (μCT, BV)
2 [27]	Hudieb et al.	SD	11–12 w	6	2	12	3 mm	8 w	N	78.12 (e) (μCT, BV/TV)	
3 [28]	Diker et al.	SD	10 w	7	2	7	2.7 mm	30 d	N	27.5 ± 6.7 (f) (SD) (H, BS/TS)	
4 [29]	Nunes et al.	W	3 m	5	1	5	4.1 mm	60 d	N	39.6 ± 27.8 (f) (SD) (H, BS/TS)	
5 [30]	Caliaperoumal et al.	Z	16 w	6	3	6	2 mm|4 mm	8 w	N/A	31.8 ± 17.4 (e) (SEM) (2 mm) (μCT, BS/TS)|96.1 ± 2.0 (e) (SEM) (4 mm) (μCT, BS/TS)	
6 [31]	Padilha et al.	W	15 w	5	2	5	2 mm	30 d	N	N/A	62.3 ± 3.58 (e) (SD) μm^2^ (H, neo-formed bone area)
7 [32]	Groppo et al.	W	N/A	6	2	6	3.3 mm	90 d	N	N/A	0.11 (e) mm^2^ (H, bone area), 7.2 ± 0.8 (f) (IqD) mm^3^ (CBCT, residual defect volume)
8 [33]	Takeda et al.	SD	12 w	6	2	12	3.8 mm	8 w	Described as almost filled (d)	N/A	4.9 ± 0.7 (f) (SD) mm^3^ (μCT, neo-formed bone volume)
9 [34]	Sun et al.	SD	N/A	6	1	6	4 mm	6 m	N	N/A	285 ± 31 (f) (SD) mgHA/cm^3^ (μCT, BMD)
10 [35]	de Oliveira et al.	W	60 d	5	1	5	2 mm	90 d	N	48.5 ± 16.4 (e) (SD) (H, BS/TS)	
11 [36]	Udagawa et al.	Fi	7 w	5	2 (c)	5	2.7 mm	28 d	N	43.4 (e) (μCT, BV/TV)	
12 [37]	Durão et al.	W	4 m	7	2	7	3 mm	6 m	N	67.7 ± 8.25 (e) (SD) (H, BL/TL)	
13 [38]	Hasuike et al.	Fi	12 w	9	1	9	2.7 mm	28 d	N	9.8 ± 5.7 (f) (SD) (μCT, BV/TV)	
14 [39]	Honma et al.	W	12 w	3 (a)	1	3 (a)	3.8 mm	24 w	Y	95 ± 3.7 (f) (SD) (qRA, BS/TS)	
15 [40]	Magyar et al.	SD	3 m	N/A	2	6	3 mm	12 w	N	38 ± 7 (e) (SE) (μCT, BV/TV)	2.2 ± 0.4 (f) (SE) mm^3^ (μCT, BV)
16 [41]	da Silva et al.	W	8 w	5	3	5	3 mm	24 w	N	18.2 (e) (H, BS/TS)	
17 [42]	Develioglu et al.	W	N/A	15	2	15	3 mm	30 d	N	N/A	Semiquantitative method (H)
18 [43]	Gurgel et al.	W	N/A	9	1	9	4 mm	45 d	N	40.25 ± 13.75 (e) (SD) (H, BL/TL)	
19 [44]	Odstrcil et al.	SD	8 w	5	1	5	3 mm	6 w	N	46 (e) (H, BS/TS)	
20 [45]	Khadra et al.	SD	2 m	5	2	10	2.7 mm	28 d	N	N/A	Semiquantitative method (H)
21 [46]	Würzler et al.	LE	35 d	3	2	3	3 mm	21 d	N	90 ± 7 (e) (SD) (qRA, BS/TS)	
22 [47]	Deporter et al.	W	N/A	10	2	10	4 mm	1 m	N	38.7 ± 19.9 (f) (H, BS/TS)	
23 [48]	Gould et al.	SD	N/A	2	2	2	2 mm	10 w	N	N/A	Descriptive histology (H)
24 [49]	Passell et al.	W	150 d	N/A (b)	4	N/A (b)	2 mm	150 d	N	N/A	Semiquantitative method (H)

Abbreviations: W, Wistar; SD rat, Sprague–Dawley rat; Z, Zucker; Fi, Fischer; LE, Long–Evans; d, days; w, weeks; m, months; Obs., number of monitored defects, not necessarily independent animals; Time, evaluation period; Closure, complete closure as classified in the source table; H, histology or histomorphometry; CBCT, cone-beam computed tomography; μCT, micro-computed tomography; qRA, quantitative radiographic analysis; BS/TS, bone surface/total defect surface; BV/TV, bone volume/total defect volume; BL/TL, bone length/total defect length; BMD, bone mineral density; SD, standard deviation; SEM, standard error of the mean; IqD, interquartile deviation. Notes: (a) number reported as 3 or 4; (b) number not clearly reported and 3 was used in the descriptive dataset; (c) one defect was 2.7 mm and the contralateral defect was 5 mm; (d) authors described defects as almost filled with bone; (e) value reported numerically in the source; (f) value digitized from a published figure.

**Table 2:** Studies included in the micro-computed tomography review and their essential characteristics.

Study [Ref.]	Authors	Strain	Age	Rats/Time	Defects/Rat	Diameter	Time	New Bone Formation (%)	Obs.	Other Methods
1 [50]	Wang et al.	SD	8 w	2	1	9 mm	1 w 4 w	1.61 ± 0.59 (b) (SD) 18.1 ± 3.3 (b) (SD)	2	H
2 [51]	Cunha et al.	W	16 w	10	1	5 mm	6 w	6.61 ± 0.97 (a) (SD)	10	H
3 [52]	Taşdemir et al.	W	N/A	6	1	6 mm	1 w 4 w	0.61 ± 0.79 (a) (SD) 8.70 ± 4.30 (a) (SD)	6	H, I
4 [53]	Prisinoto et al.	H	16 w	7	1	5 mm	2 w 8 w	13.51 ± 7.30 (a) (SD) 12.97 ± 3.30 (a) (SD)	7	H
5 [54]	Naudot et al.	SD	8 w	22	2	5 mm	2 w 4 w 6 w 8 w	29.5 ± 8.7 (a) (SD) 55.4 ± 13.9 (a) (SD) 70.3 ± 15.7 (a) (SD) 74.4 ± 14.7 (a) (SD)	22	/
6 [55]	Honda et al.	SD	8 w	2	1	9 mm	1 w 4 w	4.17 ± 2.25 (b) (SD) 14.8 ± 8.9 (b) (SD)	2	H
7 [56]	El-Fiqi et al.	N/A	N/A	N/A	2	5 mm	6 w	6.45 ± 1.43 (b) (SD)	1	H
8 [57]	Liu et al.	SD	9 w	1–2	2	6 mm	4 w 8 w	3.2 ± 1 (b) (SD) 9.0 ± 4.6 (b) (SD)	1	H
9 [58]	Kong et al.	SD	8 w	4	1	5 mm	4 w 8 w	3.28 ± 0.73 (b) (SD) 7.32 ± 0.25 (b) (SD)	4	H
10 [59]	Huang et al.	SD	Adult	4 3	1	8 mm	8 w 12 w	14.5 ± 24.5 (b) (d) 19.5 ± 6.1 (b) (d)	4 3	H, EM, CBCT
11 [60]	Souza et al.	W	N/A	12	1	5 mm	6 w	26.31 ± 8.28 (a) (SD)	12	H
12 [61]	Pinotti et al.	H	3 m	5	1	5 mm	3 d 7 d 15 d 30 d	0 0.80 ± 1.50 (b) (SD) 12.91 ± 6.71 (a) (SD) 15.56 ± 3.57 (a) (SD)	5	H, I
13 [30]	Caliaperoumal et al.	Z	16 w	6	1	8 mm	8 w	17.11 ± 2.63 (b) (SEM)	6	/
14 [62]	Honda et al.	SD	8 w	5	1	9 mm	4 w	12.7 ± 2.67 (b) (SD)	5	H, I, EM
15 [63]	Weng et al.	SD	12 w	6	1	8 mm	4 w 8 w	22.68 ± 4.54 (c) (SD) 25.40 ± 6.96 (c) (SD)	6	H
16 [64]	Tanoue et al.	SD	6 w	2	2	6 mm	0 2 w 4 w 8 w 12 w	1.30 ± 0.94 (b) (SE) 7.55 ± 2.55 (b) (SE) 18.97 ± 4.84 (b) (SE) 22.93 ± 5.00 (b) (SE) 23.48 ± 4.95 (b) (SE)	2	H, EM; FIB-SEM tomography
17 [65]	Li et al.	W	12 w	5	1	5 mm	8 w	4.1 ± 2.0 (b) (SD)	5	H
18 [66]	Kim et al.	SD	7 w	6	1	8 mm	24 w	29.2 ± 3.7 (b) (SD)	6	H, I
19 [67]	Mi et al.	SD	8 w	5	1	8 mm	8 w	8.64 ± 4.8 (a) (SD)	5	H
20 [68]	Auersvald et al.	W	3–5 m	7	1	5 mm	60 d	9.42 ± 9.70 (a) (SD)	7	H
21 [69]	Cunniffe et al.	Fi	Adult	4	1	7 mm	4 w 8 w	0.5 ± 0.7 (b) (SD) 1.2 ± 2.0 (b) (SD)	4	H, I
22 [70]	Park et al.	SD	8 w	3	2	5 mm	4 w 8 w	7.60 ± 1.8 (a) (SD) 9.70 ± 4.0 (a) (SD)	3	H
23 [71]	Gao et al.	SD	Adult	10	1	5 mm	4 w 12 w	10.5 ± 1.1 (a) (SEM) 12.2 ± 1.3 (a) (SEM)	10	H
24 [72]	Hayashi et al.	W	Adult	8	1	3.8 mm	2 w	18.3 ± 4.9 (a) (SD)	8	H
25 [73]	Thompson et al.	Fi	N/A	4	1	7 mm	4 w 8 w	3.8 ± 2.7 (b) (SD) 9.2 ± 9.6 (b) (SD)	4	H
26 [74]	Santos et al.	W	N/A	5	1	5 mm	4 w 8 w	3.2 ± 2.6 (b) (SD) 5.9 ± 2.2 (b) (SD)	5	H
27 [75]	Rezai-Rad et al.	SD	5 m	1	2	5 mm	4 w 8 w	0 0	1	H
28 [76]	Ren et al.	SD	2 w	2	1	5 mm	4 w 12 w	16.6 ± 0.65 (b) (SD) 22.1 ± 3.9 (b) (SD)	2	H, I
29 [77]	Horváthy et al.	W	N/A	5	2	4 mm	26 w	7.00 ± 4.84 (b) (SEM)	5	/
30 [78]	Kan et al.	W	N/A	5	1	5 mm	5 d 15 d 30 d	11.3 ± 1.84 (a) (SD) 13.4 ± 2.59 (a) (SD) 21.9 ± 6.49 (a) (SD)	5	H
31 [79]	Jang et al.	SD	8–9 w	10	1	8 mm	6 w	10.36 ± 7.45 (a) (d)	10	H
32 [80]	Quinlan et al.	W	N/A	8	1	7 mm	8 w	2.1 ± 0.7 (b) (SEM)	8	H
33 [81]	Amirian et al.	RN	9 w	3	2	5 mm	2 w 4 w	2.32 ± 0.35 (b) (SD) 3.26 ± 2.19 (b) (SD)	3	H, I
34 [82]	Pati et al.	SD	6 w	N/A	1	8 mm	8 w	6.32 ± 0.96 (b) (d)	1	H
35 [83]	Chou et al.	W	15 w	10	2	5 mm	2 w 4 w 6 w	11.93 ± 0.46 (b) (d) 20.87 ± 0.69 (b) (d) 26.15 ± 1.15 (b) (d)	20	H
36 [84]	Yanagi et al.	SD	10 w	6	1	8 mm	1 m 2 m 3 m	11.1 ± 1.42 (b) (SE) 16.3 ± 1.42 (b) (SE) 22.1 ± 2.5 (a) (SEM)	6	H
37 [85]	Kim et al.	SD	6 w	6	1	8 mm	8 w	5.6 ± 3.4 (b) (SD)	6	H
38 [86]	Song et al.	SD	15 w	6	1	8 mm	2 w 4 w 8 w	1.13 ± 0.41 (a) (SD) 2.25 ± 0.76 (a) (SD) 3.15 ± 0.51 (a) (SD)	6	H
39 [87]	Durão et al.	W	7–8 w	10	1	5 mm	1 m 3 m 6 m	5.78 ± 3.01 (b) (SD) 17.5 ± 4.33 (b) (SD) 26.6 ± 8.41 (b) (SD)	10	/
40 [88]	Jaiswal et al.	H	6–8 w	N/A	N/A	5 mm	6 w 10 w	29.9 ± 1.55 (b) (d) 34.02 ± 2.84 (b) (d)	1	H
41 [89]	Go et al.	SD	7 w	5	1	8 mm	4 w 8 w	2.11 ± 2.00 (a) (SD) 10.77 ± 4.47 (a) (SD)	5	H
42 [90]	Tan et al.	SD	2 m	3	1	8 mm	4 w 8 w	1.1 ± 0.6 (a) (SD) 1.4 ± 0.5 (a) (SD)	3	H
43 [38]	Hasuike et al.	Fi	12 w	9	1	2.7 mm	1 w 2 w 3 w 4 w	2.17 ± 2.49 (b) (SD) 3.64 ± 2.59 (b) (SD) 5.97 ± 3.29 (b) (SD) 9.87 (a) ± 5.56 (c) (SD)	9	H
44 [91]	Terella et al.	SD	10–11 w	6	1	8 mm	4 w 8 w	32 ± 10.57 (a) (SE) 50.0 ± 5.63 (a) (SE)	6	H
45 [92]	Bodde et al.	SD	8 w	6	1	8 mm	4 w 12 w	5.3 ± 2.0 (a) (SD) 9.8 ± 3.4 (a) (SD)	6	H, S

Abbreviations: SD, Sprague–Dawley; W, Wistar; Z, Zucker; Fi, Fischer; H and RN, strain abbreviations as reported in the source; w, weeks; m, months; d, days; Time, evaluation period; Obs., number of monitored defects, not necessarily independent animals; H, histology or histomorphometry; I, immunohistochemistry; EM, electron microscopy; CBCT, cone-beam computed tomography; FIB-SEM, focused ion beam scanning electron microscopy; S, scintigraphy; SD, standard deviation; SE(M), standard error (of the mean). Data source notes: (a) value reported numerically in the source; (b) value digitized from a published figure; (c) digitized value confirmed by the author; (d) variability measure could not be determined. Data provenance at study level: 27/45 (60.0%) included digitization; 18/45 (40.0%) were directly reported only.

## 3. Results

### 3.1. Descriptive Analysis of Subcritical-Size Defect Healing and μCT Use

Results of the review of 24 studies evaluating the success of subcritical-sized defect bone healing are listed in Table 1, along with the essential study parameters (defect diameter, evaluation period, evaluation method). It is of note that only five studies of the subcritical-size review used μCT as a methodology to follow bone defect healing [27,30,36,38,40]. None of the studies reported on the complete (100%) bony healing of a subcritical-sized defect. Only three studies reported close-to-complete bony healing (% of new bone formation ≥ 90%) [30,39,46]. Caliaperoumal et al. [30] reported a 96% bone healing of 4 mm defects after 8 weeks. Observations were made by μCT; however, only BS/TS (bone surface/total defect surface) was measured. Honma et al. [39] found a 95% bone healing of 3.8 mm defects at 24 weeks. However, their observations were made by quantitative radiographic analysis (qRA), where they measured radiopacity within the trephination defect by microradiography unit and calculated the bone-healing percentage (%) as the radiopaque area/total area of the defect. The third study by Würzler et al. [46] reported a 90% bone healing of 3 mm defects after 21 days. Their method of measuring bone healing was analogous to that of Honma et al., notably reporting BS/TS by measuring radiopacity. Additionally, Takeda and coworkers [33] evaluated the healing of 3.8 mm diameter bone defects by using the μCT methodology and described that the “defects were almost filled with bone”. However, they did not report measurements (% of bony healing) in their results. Overall, 37.5% (9/24) of studies did not report the % of new bone formation in numerals; most of these studies reported another parameter of bony healing (e.g., bone volume (BV), bone mineral density (BMD)). Of the 15 studies reporting numerical values of bone healing, 10 studies list their values numerically, and the values of the remaining five studies were retrieved by graphical analysis. Of the studies that reported the % of new bone formation, only one-third (5/15) reached a ≥ 50% margin of bony healing. Most (14/15), however, reported a ≥ 25% bone defect healing closure.

While performing the μCT review, three studies with defect sizes of < 5 mm were identified [38,72,77]. One of them was already included in the subcritical-size review [38], and the results of the remaining two are analyzed here separately [72,77]. Hayashi et al. [72] created 3.8 mm defects and achieved 18.3% healing in a two-week observation period, and Horváthy et al. [77] created 4 mm defects and achieved 7% healing in 26 weeks.

Results of the literature review of the use of the μCT methodology to evaluate bone healing in the rat calvaria defect model are listed in Table 2, along with the essential study parameters (defect diameter, evaluation period, evaluation method) and success of bony healing. Forty-five studies were included in the final review, while 97 studies were excluded from the review. Forty-eight studies were excluded because they did not include numerical μCT results (BV ± SD), 34 because they implanted material into “control” defects (most commonly resorbable collagen sponges), and 15 because of alternative study protocols (most commonly the bone augmentation model). In none of the 45 studies did calvaria bone defect healing reach complete (or ≥ 90%) defect closure, and only four percent (2/45) [54,91] made the ≥50% margin of bone regeneration. Naudot et al. [54] presented the successful healing of 5 mm bony defects in a study including a high number of observations (N = 22), which gives credibility to the result. After 2 weeks, their defects were already 29.5% healed, reaching the > 50% margin by 4 weeks (55.4%) and finally achieving 74.4% at 8 weeks, which is the best spontaneous healing we observed in the μCT review. The second study, that by Terella et al. [91], reached 50% closing of 8 mm defects in eight weeks of observation, which is closer to the result of the majority of studies. The majority of studies (49%, 22/45) remained in the 0–10% interval, and another 24% (11/45) achieved the 10–20% interval. There was an additional 20% (9/45) of studies in the 20–30% interval. Altogether, only three studies (7%) [54,88,91] exceeded 30% of bony defect healing.

One of the benefits of μCT is the possibility of in vivo measurement, which enables researchers to follow bone healing longitudinally along the life span of one animal. Eleven percent (5/45) of studies in our review used the option of in vivo measurement [38,54,64,83,91]. Of these five studies, two used in vivo μCT within three time points [64,91] and three studies within four time points [38,54,83].

Of the 45 studies included in the review, 43 complemented the use of μCT with descriptive histology; however, only 19 studies quantified the histological results (histomorphometry). Of the 45 included μCT studies, 27 (60.0%) required digitization of at least one mean or variability value from published graphs, whereas 18 (40.0%) exclusively provided directly reported numerical values.

### 3.2. Exploratory Grouped Summaries of μCT Outcomes

Study-level data are presented in Tables S1 and S2. Across the best represented groups, healing generally increased with longer observation, and 5 mm defects tended to show greater proportional healing than 8 mm defects at comparable times. Wide ranges within each group indicate that defect size and duration alone do not explain the variation. Because several values were digitized or based on uncertain variance information, these summaries are descriptive and should not be interpreted as pooled benchmarks.

## 4. Discussion

The rat calvarial critical-size defect model remains an important tool in bone-healing and cranio-maxillofacial regeneration research. Its value lies in the creation of a reproducible bone defect in which spontaneous repair is sufficiently limited to allow investigators to evaluate whether an intervention meaningfully improves regeneration. In agreement with Vajgel et al. [23], the present review supports the practical use of the 5 mm rat calvarial defect as a critical-size model. However, the findings also indicate that the distinction between critical- and subcritical-size defects should be interpreted cautiously. Critical size should be understood as an operational experimental concept rather than a fixed anatomical constant. It depends not only on defect diameter but also on the duration of observation, animal characteristics, surgical technique, local biology, and method used to define successful healing. Examples include the testing of growth-factor delivery, cell-based constructs, and scaffolds intended to improve craniofacial bone repair [59,73,80]. This provides preclinical evidence for selecting and refining candidate treatments. However, the untreated-control outcomes assessed here cannot demonstrate clinical benefit, and differences in anatomy, loading, scale, and host biology limit direct extrapolation to patients.

Importantly, the significance of this model should not be framed solely in terms of generating evidence for human therapies. The literature reviewed here also addresses an animal research question in its own right: how the defect size, observation period, surgical technique, and outcome definition influence the burden, informativeness, and reproducibility of a commonly used rat model. Establishing these relationships is a form of refinement. It allows investigators and ethics committees to select the least burdensome design capable of answering the research question, to avoid experiments based on the incorrect assumption that any defect smaller than 5 mm will predictably self-heal, and to reduce repetition of poorly comparable studies.

The most important message from the sub-5 mm literature is that the term “subcritical” should not be equated with predictable or complete spontaneous healing. Across the 24 studies included in the subcritical-size review, complete bony healing was not robustly documented. Only three studies reported close-to-complete healing of 90% or more [30,39,46], and these reports used different outcome definitions and measurement techniques. Caliaperoumal et al. [30] reported the high closure of 4 mm defects using μCT-based BS/TS, whereas Honma et al. [39] and Würzler et al. [46] relied on radiographic or radiopacity-based surface measures. These findings suggest that substantial closure can occur in some smaller defects, but they also illustrate why the endpoint must be interpreted in relation to the measurement method. Surface closure, radiopacity, bone length, and volumetric restoration are not interchangeable.

The evidence for incomplete spontaneous healing is especially clear in the older histological literature. Passell et al. [49] observed that even 2 mm calvarial defects were not bridged by bone after 150 days. Histological examination showed marginal bone apposition with a fibrous scar remaining centrally, and cellular activity had largely ceased by the end of the observation period. This observation supports the idea that spontaneous calvarial healing may be limited even in small defects. Nevertheless, it would be too strong to conclude that all sub-5 mm defects are “critical” in the same way as a 5 mm or larger defect. Smaller defects may be biologically more permissive than larger defects but still fail to achieve reliable full closure. For experimental design, this distinction is important: a defect may be smaller than the conventionally accepted critical size and still be unsuitable for studies in which near-complete spontaneous repair is assumed. Complete closure may be unsuitable as the sole endpoint because a prespecified follow-up can end before repair is complete, while some defects may approach a persistent plateau of marginal bone formation and central fibrosis [39,49]. Extending observation could capture further repair in some settings but cannot be assumed to yield 100% closure. These are possible explanations rather than documented reasons for the endpoint choices of individual studies. Prespecified quantitative outcomes at biologically justified time points can detect partial regeneration without prolonging animal exposure solely to pursue an uncertain endpoint.

This has practical implications for the selection of defect size. Critical-size models are well suited for testing strong osteogenic or osteoconductive interventions because untreated defects generally remain insufficiently healed and treatment effects can be clearly demonstrated [4,93,94,95]. Bilateral models are particularly useful in this setting because the treated defect can be compared with an untreated or differently treated contralateral defect in the same animal. In contrast, studies evaluating physiological modifiers of bone repair, such as age, sex, systemic disease, medication effects, or subtle regenerative stimuli, may require smaller defects in which partial spontaneous healing occurs. However, in such studies, baseline healing must be measured within the experiment rather than assumed from the term “subcritical”. The present findings therefore support continued use of critical-size terminology, but only if the term is applied with clear reference to the study design, observation period, and selected healing endpoint.

From an animal welfare perspective, neither a 5 mm defect nor any larger defect should be chosen by convention alone. When a smaller defect can provide the required dynamic range, it should be preferred, provided that its spontaneous healing is characterized by a concurrent control and that sample-size planning preserves scientific validity. A bilateral design can reduce the number of animals by enabling within-animal comparisons, but it must not be adopted solely for numerical efficiency: the additional surgical burden, cumulative defect area, postoperative discomfort, and risk of complications require explicit harm–benefit justification. Reduction and refinement must therefore be considered together.

Bone organoids and 3D-bioprinted tissue constructs hold promise for replacing selected animal experiments in bone regeneration research. By reproducing aspects of bone formation and cellular interactions in vitro, these systems could enable the preliminary screening of biomaterials and regenerative treatments, reducing reliance on animal models. However, they do not yet fully reproduce the complexity of whole-organism physiology, including vascular, immune, and systemic interactions involved in bone healing.

The grouped study-level data support the same general interpretation while also showing why caution is necessary. Healing generally increased with longer observation and tended to be greater in 5 mm defects than in 8 mm defects at comparable times, but values varied widely within each group. These summaries are therefore best understood as descriptive indicators of direction and range rather than as pooled benchmarks.

Wide variation remained evident after grouping by defect diameter and observation period, showing that size and healing time do not explain reported outcomes alone. Likely contributors include rat strain, age, sex, weight, systemic health, defect number and location, periosteal preservation, dural integrity, drilling technique, irrigation, local thermal injury, postoperative conditions, and μCT acquisition and analysis settings. Historical control values therefore cannot reliably replace internal controls. Underreporting also has an ethical consequence because animals may be exposed to procedures without generating reliable, reusable knowledge; model and reporting standardization is a refinement measure as well as a technical priority.

A major contribution of μCT is that it allows for the three-dimensional quantification of new bone formation. Compared with descriptive histology or semiquantitative radiographic methods, μCT provides more precise information on bone volume, mineralized tissue distribution, and defect architecture. It can also be performed in vivo, allowing for the longitudinal follow-up of the same animal and reducing inter-animal variability [22]. In the present review, however, only 5 of 45 μCT-based studies used in vivo measurements [38,54,64,83,91]. This shows that the longitudinal potential of μCT remains underused. Greater use of standardized in vivo μCT protocols could improve understanding of the time course of calvarial healing, but this would require careful attention to radiation exposure, scan resolution, and consistency of repeated region-of-interest placement. When longitudinal scanning replaces separate terminal cohorts, it may reduce animal numbers and increase the information obtained from each animal. Nevertheless, repeated anesthesia, handling, and exposure to ionizing radiation are additional burdens, scan frequency and dose should be minimized and justified, and ex vivo imaging may remain preferable when repeated in vivo imaging is not necessary for the scientific objective.

At the same time, μCT has not solved the problem of comparability between studies. Greater measurement precision within a study does not automatically produce comparability across studies. The reviewed literature showed considerable variation in how μCT outcomes were acquired, processed, and reported. In many studies, numerical data were not reported in a directly reusable form, and some values had to be recovered from figures. Forty-eight potentially relevant μCT studies were excluded because numerical μCT results were not available. If μCT data are presented only graphically or without clear definitions of BV, TV, thresholding, and the region of interest, the results cannot be meaningfully compared or reused. Providing numerical results in tables or accessible datasets, including outcomes from untreated and negative-control defects, would increase the information yield of each experiment and reduce the avoidable duplication of animal use. Failure to report such data weakens both the scientific value and ethical justification of the original animal work.

The historical evolution of outcome assessment further complicates interpretation. Earlier studies relied on descriptive histology, semiquantitative histological evaluation, or radiographic opacity [45,48,49]. Later, histomorphometry and radiographic densitometry became more common, while the use of CBCT or μCT has recently been increasing [32,38,40]. Correlations between μCT and histomorphometry have been reported [96,97,98,99], but the absolute values obtained by these methods cannot be assumed to be directly interchangeable. Histology remains valuable because it identifies tissue type, fibrous tissue, inflammation, vascularity, and bone maturity, while μCT primarily detects mineralized tissue. The two methods should therefore be considered complementary rather than competing. μCT is well suited as the principal quantitative tool, whereas histology and histomorphometry provide biological validation of the μCT signal.

The subcritical-size review also illustrates the limits of comparing older and newer measurement approaches. Only a minority of studies used μCT, while many relied on histology, histomorphometry, radiographic density, or semiquantitative observations. Quantitative aggregation was therefore not appropriate. The evidence nevertheless shows that smaller rat calvarial defects often undergo only partial repair and does not support the assumption that defects smaller than 5 mm reliably heal to completion.

The manner in which healing is expressed is another central issue. BV/TV is currently the most useful and most commonly transferable μCT-derived parameter because it expresses the proportion of mineralized new bone within a defined total defect volume. However, BV/TV alone does not fully describe defect repair. Bone volume and defect bridging are related but not identical. A defect may contain a substantial amount of new bone at the margins while still lacking a continuous bridge across the center. Conversely, a thin bridge may restore continuity but represent only a small fraction of the original calvarial volume. For the calvarium, which functions as a protective bony plate, both volume and continuity are relevant. Therefore, when authors describe complete healing or successful bridging, BV/TV should be complemented by a bridging-related or surface-related measure, such as BS/TS, the defect closure area, or another clearly defined metric.

This distinction is particularly important when interpreting studies that report near-complete healing. Some authors describe defects as almost completely healed [33,39], but such descriptions are difficult to compare unless supported by quantitative measures. In our view, claims of complete or near-complete bony healing should be supported by both a high volumetric value and a high bridging or surface value. A high BS/TS value may indicate surface coverage or bridging, but it does not necessarily prove restoration of the original three-dimensional bone volume. Similarly, a high BV/TV value may indicate substantial mineralized tissue formation, but it may not prove continuous bridging. Reporting both types of outcomes would make conclusions about healing more transparent and comparable.

The proposed minimum reporting standards for future rat calvarial μCT studies are summarized in Table 3. In addition to BV/TV and an explicitly defined bridging measure, mandatory reporting should include the nominal isotropic voxel size in μm, effective spatial resolution when characterized, and exact three-dimensional volume of interest (VOI): shape, diameter or dimensions, height or slice range, anatomical position, and treatment of pre-existing cortical margins. Voxel size should not be equated with measured spatial resolution. Phantom composition, hydroxyapatite concentrations in mg HA/cm^3^, and calibration procedure should be reported, or it should be explicitly stated that calibration was not performed and the implications for density-based outcomes and thresholds explained [22].

Prospective ethical approval is required for all new studies using live rats, together with evidence that existing data or non-animal methods cannot answer the question. Protocols should justify the defect, animal numbers, and time points; appropriate anesthesia, analgesia, monitoring, and humane endpoints should be provided; and prompt intervention when welfare thresholds are reached should be ensured. Approval alone does not demonstrate refinement, and avoidable pain or suffering is unacceptable.

The wide between-study variation supports contemporaneous internal controls, but these controls should be designed efficiently. Historical values cannot reliably replace a control group, although the present review can inform plausible healing ranges for prospective sample-size calculations. A single well-characterized control group may be shared across compatible intervention arms when prospectively planned and statistically valid. Bilateral or repeated-measures designs may further reduce animal numbers if the experimental unit is defined correctly, pseudoreplication is avoided, and the additional burden is justified. Before any new animal work, investigators should consider whether literature synthesis, archived data or images, in vitro or ex vivo systems, or computational approaches can answer the question. Replacement can be advanced by using three-dimensional cell cultures, bone organoids, and printed or bioprinted in vitro constructs to screen material compatibility, mineralization, and candidate treatments before animal testing. Recent vascularized and mineralized organoid work illustrates not only progress but also continued reliance on animal-derived components and in vivo maturation or validation [100]. Likewise, 3D-printed scaffolds implanted in rats [59,82] are not themselves replacements for animal experiments. We therefore anticipate a role for these approaches in replacing selected screening experiments, while complete replacement for integrated vascular, immune, and long-term host responses remains to be demonstrated. This review did not quantify a field-wide trend in animal use.

Although Cooper et al. [101] questioned the practical value of the critical-size concept because smaller defects may also fail to heal, the present review supports retaining it as an operational study design tool rather than a universal biological rule. A 5 mm defect remains a defensible practical benchmark; smaller defects should be defined by observed baseline healing, and investigators should select the least severe defect that provides an adequate window for the prespecified endpoint.

Taken together, the evidence supports the 5 mm rat calvarial defect as a practical critical-size model while showing that smaller defects cannot be assumed to heal completely. Its main contribution is model refinement: studies should proceed only when existing or non-animal evidence is insufficient, use the least burdensome valid design, obtain prior ethical approval, and report standardized μCT methods and reusable data. These measures can improve reproducibility and reduce unnecessary animal use and suffering.

This review has several limitations. It was not prospectively registered, used a single database, and did not apply a formal risk-of-bias tool. The included studies varied substantially in animal characteristics, surgeries, observation periods, and outcome reporting, and some numerical values had to be digitized when author confirmation was unavailable. The grouped study-level summaries are therefore descriptive. In addition, animal welfare variables were not extracted systematically, so inclusion should not be interpreted as evidence that every primary study met current ethical and reporting standards. Relevant biomaterials and tissue-engineering studies indexed in Scopus, Web of Science, or other sources may have been missed. The search was not extended because of practical time and resource constraints rather than because of evidence that other databases would add no information. We expect the qualitative emphasis on endpoint definition and transparent μCT reporting to remain relevant, but the effect of a broader search on study counts, healing distributions, and defect-size conclusions is unknown. The PubMed subset therefore cannot define universal baseline healing rates.

## 5. Conclusions

Within the PubMed-indexed literature reviewed here, the evidence supports the 5 mm rat calvarial defect as a practical critical-size model, but smaller defects also heal incompletely and variably. Defect classification should therefore be tied to the animal characteristics, observation time, technique, and prespecified endpoint. Future studies should report μCT-derived BV/TV, add a bridging measure when relevant, describe acquisition and analysis settings fully, and provide reusable numerical control data. Replacement, reduction, and refinement means using prior evidence and non-animal methods should be applied where possible; the least severe valid defect should be selected; animal numbers, controls, and imaging schedules should be prospectively justified; and appropriate ethical approval, anesthesia, analgesia, monitoring, and humane endpoints should be ensured. These practices can improve reproducibility while minimizing animal use and burden.

## Figures and Tables

**Table 3:** Minimum reporting standards for future rat calvarial μCT studies.

Domain	Minimum Reporting Items
Animals and model	Strain, age, sex, weight, systemic condition, animal and defect numbers, unilateral/bilateral design, exact defect diameter and location, observation period, and control status or implanted material
Experimental design	Sample-size justification, experimental unit, randomization, blinding, and rationale for animal characteristics, defect size and number, and time points
Surgery	Trephine/drill method, irrigation, periosteal handling, dural integrity, and complication management
Ethics and welfare	Approving body, project code/date, regulatory framework, anesthesia and analgesia, housing, monitoring, humane endpoints, adverse events, exclusions, mortality, and euthanasia
μCT acquisition	Scanner; nominal voxel size (μm) and measured spatial resolution; voltage/current/filter; reconstruction; segmentation; phantom material, HA concentrations (mg HA/cm^3^), calibration procedure or explicit non-use; exact volume-of-interest (VOI) geometry and dimensions; radiation dose/schedule for repeated scans
Outcomes and validation	BV/TV (plus absolute BV where useful), a bridging metric such as BS/TS when relevant, and histology when material or tissue characteristics complicate μCT interpretation

## Data Availability

The study-level data supporting this review are provided in the article and Supplementary Materials. Additional extraction files are available from the corresponding author upon reasonable request.

## Supplementary Materials

The following supporting information can be downloaded at: https://www.mdpi.com/article/10.3390/ani16193142/s1, Table S1: Study-level data for the best represented 5 and 8 mm defect groups; Table S2: Full study-level dataset grouped by defect size and observation period. The supplementary tables cite studies included on the main reference list.
