# Supplementary material for: Rat Calvarial Defect Models in Bone Regeneration Research: A Narrative Review of Critical Size and Micro-Computed Tomography Outcome Reporting
Source: Animals (Basel). 2026 Oct 7;16(19):3142. doi: 10.3390/ani16193142 (PMC13663530; doi:10.3390/ani16193142)
Supplement: Supplementary file 1 [file animals-16-03142-s001.zip › Supplementary Table S1.pdf]

Table S1. Study-level data for the best represented 5 and 8 mm defect groups.

Study [ref.]	Author	Defect diameter (mm)	Observations	Evaluation period	Reported new bone formation (%)	Mean (%)	SEM (%)
5 mm							
Observation period: 4w							
1 [54]	Naudot	5	22	4	55.4 ± 13.9(a) (SD)	55.4	2.96
2 [58]	Kong	5	4	4	3.28 ± 0.73(b) (SD)	3.28	0.37
3 [61]	Pinotti	5	5	30d	15.56 ± 3.57(a) (SD)	15.56	1.60
4 [70]	Park	5	3	4	7.60 ± 1.8(a) (SD)	7.60	1.04
5 [71]	Gao	5	10	4	10.5 ± 1.1(a) (SEM)	10.5	1.10
6 [74]	Santos	5	5	4	3.2 ± 2.6(b) (SD)	3.2	1.16
7 [75]	Rezai-Rad	5	1	4	0.00 ± 0.00(a) (SEM)	0.00	0.00
8 [76]	Ren	5	2	4	16.6 ± 0.65(b) (SD)	16.6	0.46
9 [78]	Kan	5	5	30d	21.9 ± 6.49(a) (SD)	21.9	2.90
10 [81]	Amirian	5	3	4	3.26 ± 2.19(b) (SD)	3.26	1.26
11 [83]	Chou	5	20	4	20.87 ± 0.69(b) (d)	20.87	0.69
12 [87]	Durão	5	10	1m	5.78 ± 3.01(b) (SD)	5.78	0.95
Observation period: 8w							
1 [53]	Prisinoto	5	7	8	12.97 ± 3.30(a) (SD)	12.97	1.25
2 [54]	Naudot	5	22	8	74.4 ± 14.7(a) (SD)	74.4	3.13
3 [58]	Kong	5	4	8	7.32 ± 0.25(b) (SD)	7.32	0.13
4 [65]	Li	5	5	8	4.1 ± 2.0(b) (SD)	4.1	0.89
5 [68]	Auersvald	5	7	60d	9.42 ± 9.70(a) (SD)	9.42	3.67
6 [70]	Park	5	3	8	9.70 ± 4.0(a) (SD)	9.70	2.31
7 [74]	Santos	5	5	8	5.9 ± 2.2(b) (SD)	5.9	0.98
8 [75]	Rezai-Rad	5	1	8	0.00 ± 0.00(a) (SEM)	0.00	0.00
8 mm							
Observation period: 4w							
1 [63]	Weng	8	6	4	22.68 ± 4.54(c) (SD)	22.68	1.85
2 [84]	Yanagi	8	6	1m	11.1 ± 1.42(b) (SE)	11.1	1.42
3 [86]	Song	8	6	4	2.25 ± 0.76(a) (SD)	2.25	0.31
4 [89]	Go	8	5	4	2.11 ± 2.00(a) (SD)	2.11	0.89
5 [90]	Tan	8	3	4	1.1 ± 0.6(a) (SD)	1.1	0.35
6 [91]	Terella	8	6	4	32 ± 10.57(a) (SE)	32	10.57
7 [92]	Bodde	8	6	4	5.3 ± 2.0(a) (SD)	5.3	0.82
Observation period: 8w							
1 [59]	Huang	8	4	8	14.5 ± 24.5(b) (d)	14.5	24.5
2 [30]	Caliaperoumal	8	6	8	17.11 ± 2.63(b) (SEM)	17.11	2.63
3 [63]	Weng	8	6	8	25.40 ± 6.96(c) (SD)	25.40	2.84
4 [67]	Mi	8	5	8	8.64 ± 4.8(a) (SD)	8.64	2.15
5 [82]	Pati	8	1	8	6.32 ± 0.96(b) (d)	6.32	0.96
6 [84]	Yanagi	8	6	2m	16.3 ± 1.42(b) (SE)	16.3	1.42
7 [66]	Kim	8	6	8	5.6 ± 3.4(b) (SD)	5.6	1.39
8 [86]	Song	8	6	8	3.15 ± 0.51(a) (SD)	3.15	0.21
9 [89]	Go	8	5	8	10.77 ± 4.47(a) (SD)	10.77	2.00
10 [90]	Tan	8	3	8	1.4 ± 0.5(a) (SD)	1.4	0.29
11 [91]	Terella	8	6	8	50 ± 5.63(a) (SE)	50	5.63

Abbreviations: d, day(s); w, week(s); m, month(s); SD, standard deviation; SE(M), standard error (of the mean). Data-source notes: (a) value reported numerically in the source; (b) value digitized from a published figure; (c) digitized value confirmed by the author; (d) variability measure could not be determined. The table presents study-level values only; no pooled estimates are reported.
