# Supplementary material for: Rat Calvarial Defect Models in Bone Regeneration Research: A Narrative Review of Critical Size and Micro-Computed Tomography Outcome Reporting
Source: Animals (Basel). 2026 Oct 7;16(19):3142. doi: 10.3390/ani16193142 (PMC13663530; doi:10.3390/ani16193142)
Supplement: Supplementary file 1 [file animals-16-03142-s001.zip › Supplementary Table S2.pdf]

Table S2. Full study-level dataset grouped by defect size and observation period.

Study [ref.]	Author	Defect diameter (mm)	Observations	Evaluation period	Reported new bone formation (%)	Mean (%)	SEM (%)
<5 mm defects							
Observation period: 1w							
1 [38]	Hasuike	2.7	9	1	2.17 ± 2.49(b) (SD)	2.17	0.83
Observation period: 2w							
1 [38]	Hasuike	2.7	9	2	3.64 ± 2.59(b) (SD)	3.64	0.86
2 [72]	Hayashi	3.8	8	2	18.3 ± 4.9(a) (SD)	18.3	1.73
Observation period: 3w							
1 [38]	Hasuike	2.7	9	3	5.97 ± 3.29(b) (SD)	5.97	1.10
Observation period: 4w							
1 [38]	Hasuike	2.7	9	4	9.87(a) ± 5.56(c) (SD)	9.87	1.85
Observation period: 20w-30w							
1 [77]	Horváthy	4	5	26w	7.00 ± 4.84(b) (SEM)	7.00	4.84
5 mm							
Observation period: 1w							
1 [61]	Pinotti	5	5	7d	0.80 ± 1.50(b) (SD)	0.80	0.67
2 [78]	Kan	5	5	5d	11.3 ± 1.84(a) (SD)	11.3	0.82
Observation period: 2w							
1 [53]	Prisinoto	5	7	2	13.51 ± 7.30(a) (SD)	13.51	2.76
2 [54]	Naudot	5	22	2	29.5 ± 8.7(a) (SD)	29.5	1.85
3 [61]	Pinotti	5	5	15d	12.91 ± 6.71(a) (SD)	12.91	3.00
4 [78]	Kan	5	5	15d	13.4 ± 2.59(a) (SD)	13.4	1.16
5 [81]	Amirian	5	3	2	2.32 ± 0.35(b) (SD)	2.32	0.20
6 [83]	Chou	5	20	2	11.93 ± 0.46(b) (d)	11.93	0.46
Observation period: 4w							
1 [54]	Naudot	5	22	4	55.4 ± 13.9(a) (SD)	55.4	2.96
2 [58]	Kong	5	4	4	3.28 ± 0.73(b) (SD)	3.28	0.37
3 [61]	Pinotti	5	5	30d	15.56 ± 3.57(a) (SD)	15.56	1.60
4 [70]	Park	5	3	4	7.60 ± 1.8(a) (SD)	7.60	1.04
5 [71]	Gao	5	10	4	10.5 ± 1.1(a) (SEM)	10.5	1.10
6 [74]	Santos	5	5	4	3.2 ± 2.6(b) (SD)	3.2	1.16
7 [75]	Rezai-Rad	5	1	4	0.00 ± 0.00(a) (SEM)	0.00	0.00
8 [76]	Ren	5	2	4	16.6 ± 0.65(b) (SD)	16.6	0.46
9 [78]	Kan	5	5	30d	21.9 ± 6.49(a) (SD)	21.9	2.90
10 [81]	Amirian	5	3	4	3.26 ± 2.19(b) (SD)	3.26	1.26
11 [83]	Chou	5	20	4	20.87 ± 0.69(b) (d)	20.87	0.69
12 [87]	Durão	5	10	1m	5.78 ± 3.01(b) (SD)	5.78	0.95
Observation period: 6w							
1 [54]	Naudot	5	22	6	70.3 ± 15.7(a) (SD)	70.3	3.35
2 [51]	Cunha	5	10	6	6.61 ± 0.97(a) (SD)	6.61	0.31
3 [56]	El-Fiqi	5	1	6	6.45 ± 1.43(b) (SD)	6.45	1.43
4 [60]	Souza	5	12	6	26.31 ± 8.28(a) (SD)	26.31	2.39
5 [83]	Chou	5	20	6	26.15 ± 1.15(b) (d)	26.15	1.15
6 [88]	Jaiswal	5	1	6	29.9 ± 1.55(b) (d)	29.9	1.55

Table S2. Continued (part 2 of 3).

Study [ref.]	Author	Defect diameter (mm)	Observations	Evaluation period	Reported new bone formation (%)	Mean (%)	SEM (%)
**Observation period: 8w**							
1 [53]	Prisinoto	5	7	8	12.97 ± 3.30(a) (SD)	12.97	1.25
2 [54]	Naudot	5	22	8	74.4 ± 14.7(a) (SD)	74.4	3.13
3 [58]	Kong	5	4	8	7.32 ± 0.25(b) (SD)	7.32	0.13
4 [65]	Li	5	5	8	4.1 ± 2.0(b) (SD)	4.1	0.89
5 [68]	Auersvald	5	7	60d	9.42 ± 9.70(a) (SD)	9.42	3.67
6 [70]	Park	5	3	8	9.70 ± 4.0(a) (SD)	9.70	2.31
7 [74]	Santos	5	5	8	5.9 ± 2.2(b) (SD)	5.9	0.98
8 [75]	Rezai-Rad	5	1	8	0.00 ± 0.00(a) (SEM)	0.00	0.00
**Observation period: 10w**							
1 [88]	Jaiswal	5	1	10	34.02 ± 2.84(b) (d)	34.02	2.84
**Observation period: 12w**							
1 [71]	Gao	5	10	12	12.2 ± 1.3(a) (SEM)	12.2	1.30
2 [76]	Ren	5	2	12	22.1 ± 3.9(b) (SD)	22.1	2.76
3 [87]	Durão	5	10	3m	17.5 ± 4.33(b) (SD)	17.5	1.37
**Observation period: 20-30w**							
1 [87]	Durão	5	10	6m	26.6 ± 8.41(b) (SD)	26.6	2.66
**6 mm**							
**Observation period: 1w**							
1 [52]	Taşdemir	6	6	1	0.61 ± 0.79(a) (SD)	0.61	0.32
**Observation period: 2w**							
1 [64]	Tanoue	6	2	2	7.55 ± 2.55(b) (SE)	7.55	2.55
**Observation period: 4w**							
1 [52]	Taşdemir	6	6	4	8.70 ± 4.30(a) (SD)	8.70	1.76
2 [57]	Liu	6	1	4	3.2 ± 1(b) (SD)	3.2	0.58
3 [64]	Tanoue	6	2	4	18.97 ± 4.84(b) (SE)	18.97	4.84
**Observation period: 8w**							
1 [57]	Liu	6	1	8	9.0 ± 4.6(b) (SD)	9.0	2.66
2 [64]	Tanoue	6	2	8	22.93 ± 5.00(b) (SE)	22.93	5.00
**Observation period: 12w**							
1 [64]	Tanoue	6	2	12	23.48 ± 4.95(b) (SE)	23.48	4.95
**7 mm**							
**Observation period: 4w**							
1 [69]	Cunniffe	7	4	4	0.5 ± 0.7(b) (SD)	0.5	0.35
2 [73]	Thompson	7	4	4	3.8 ± 2.7(b) (SD)	3.8	1.35
**Observation period: 8w**							
1 [69]	Cunniffe	7	4	8	1.2 ± 2.0(b) (SD)	1.2	1.00
2 [73]	Thompson	7	4	8	9.2 ± 9.6(b) (SD)	9.2	4.80
3 [80]	Quinlan	7	8	8	2.1 ± 0.7(b) (SEM)	2.1	0.70

Table S2. Continued (part 3 of 3).

Study [ref.]	Author	Defect diameter (mm)	Observations	Evaluation period	Reported new bone formation (%)	Mean (%)	SEM (%)
**8 mm**							
**Observation period: 2w**							
1 [86]	Song	8	6	2	1.13 ± 0.41(a) (SD)	1.13	0.17
**Observation period: 4w**							
1 [63]	Weng	8	6	4	22.68 ± 4.54(c) (SD)	22.68	1.85
2 [84]	Yanagi	8	6	1m	11.1 ± 1.42(b) (SE)	11.1	1.42
3 [86]	Song	8	6	4	2.25 ± 0.76(a) (SD)	2.25	0.31
4 [89]	Go	8	5	4	2.11 ± 2.00(a) (SD)	2.11	0.89
5 [90]	Tan	8	3	4	1.1 ± 0.6(a) (SD)	1.1	0.35
6 [91]	Terella	8	6	4	32 ± 10.57(a) (SE)	32	10.57
7 [92]	Bodde	8	6	4	5.3 ± 2.0(a) (SD)	5.3	0.82
**Observation period: 6w**							
1 [79]	Jang	8	10	6	10.36 ± 7.45(a) (d)	10.36	7.45
**Observation period: 8w**							
1 [59]	Huang	8	4	8	14.5 ± 24.5(b) (d)	14.5	24.5
2 [30]	Caliaperoumal	8	6	8	17.11 ± 2.63(b) (SEM)	17.11	2.63
3 [63]	Weng	8	6	8	25.40 ± 6.96(c) (SD)	25.40	2.84
4 [67]	Mi	8	5	8	8.64 ± 4.8(a) (SD)	8.64	2.15
5 [82]	Pati	8	1	8	6.32 ± 0.96(b) (d)	6.32	0.96
6 [84]	Yanagi	8	6	2m	16.3 ± 1.42(b) (SE)	16.3	1.42
7 [66]	Kim	8	6	8	5.6 ± 3.4(b) (SD)	5.6	1.39
8 [86]	Song	8	6	8	3.15 ± 0.51(a) (SD)	3.15	0.21
9 [89]	Go	8	5	8	10.77 ± 4.47(a) (SD)	10.77	2.00
10 [90]	Tan	8	3	8	1.4 ± 0.5(a) (SD)	1.4	0.29
11 [91]	Terella	8	6	8	50 ± 5.63(a) (SE)	50	5.63
**Observation period: 12w**							
1 [59]	Huang	8	3	12	19.5 ± 6.1(b) (d)	19.5	6.1
2 [84]	Yanagi	8	6	3m	22.1 ± 2.5(a) (SEM)	22.1	2.50
3 [92]	Bodde	8	6	12	9.8 ± 3.4(a) (SD)	9.8	1.39
**Observation period: 20-30w**							
1 [66]	Kim	8	6	24	29.2 ± 3.7(b) (SD)	29.2	1.51
**9 mm**							
**Observation period: 1w**							
1 [50]	Wang	9	4	1	1.61 ± 0.59(b) (SD)	1.61	0.30
2 [55]	Honda	9	2	1	4.17 ± 2.25(b) (SD)	4.17	1.59
**Observation period: 4w**							
1 [50]	Wang	9	4	4	18.1 ± 3.3(b) (SD)	18.1	1.65
2 [55]	Honda	9	2	4	14.8 ± 8.9(b) (SD)	14.8	6.29
3 [62]	Honda	9	5	4	12.7 ± 2.67(b) (SD)	12.7	1.19

Abbreviations: d, day(s); w, week(s); m, month(s); SD, standard deviation; SE(M), standard error (of the mean). Data-source notes: (a) value reported numerically in the source; (b) value digitized from a published figure; (c) digitized value confirmed by the author; (d) variability measure could not be determined. The table presents study-level values only; no pooled estimates are reported.
